# Insufficient cerebrospinal fluid penetration of tedizolid: a case report

**DOI:** 10.1093/jacamr/dlaf100

**Published:** 2025-07-08

**Authors:** Xavier Brousse, Minh P Lê, Marin Lahouati, Paul Petitgas

**Affiliations:** Infectious Diseases Unit, University Hospital of La Réunion, Saint-Pierre, Reunion Island 97410, France; Infectious Diseases Unit, University Hospital of Bordeaux, Bordeaux F-33000, France; Pharmacology Department, AP-HP, Bichat Claude Bernard Hospital, Paris, France; Université Paris Cité, INSERM, IAME, UMR 1137, Paris F-75006, France; Clinical Pharmacy Department, University Hospital of Bordeaux, Bordeaux F-33000, France; Infectious Diseases Unit, University Hospital of La Réunion, Saint-Pierre, Reunion Island 97410, France

## Abstract

**Purpose:**

Tedizolid, a novel oxazolidinone developed for the treatment of skin and soft tissue infections, exhibits a longer half-life than linezolid, with reduced myelosuppression and neurotoxicity. However, its penetration into the cerebrospinal fluid (CSF) remains poorly characterized. Our case report evaluated the CSF penetration of tedizolid.

**Case presentation:**

We present a case of parameningeal aseptic meningitis secondary to otogenic skull base osteomyelitis. Tedizolid concentrations in CSF and serum were measured using liquid chromatography and tandem mass spectrometry. The trough CSF concentration of tedizolid was below the limit of quantification, whereas the corresponding serum concentration was within the expected therapeutic range. A brief review of the literature corroborated the observation that CSF penetration of tedizolid was markedly lower than that of linezolid.

**Conclusion:**

Considering the consistently low CSF concentrations of tedizolid, linezolid remains the preferred agent for the treatment of CNS infections.

## Introduction

Tedizolid phosphate is the second oxazolidinone to become available and offers the notable advantage of once-daily dosing, attributed to its extended half-life, simplifying its administration.^1,2^ In contrast, linezolid, the first oxazolidinone approved by the U.S. Food and Drug Administration (FDA) in 2000, requires twice-daily dosing (600 mg) due to its shorter half-life of approximately 5 h. Linezolid is primarily approved for the treatment of skin and soft tissue infections (SSTI), pneumonia, and vancomycin-resistant enterococcal bloodstream infections.^3^ In contrast, tedizolid is currently approved only for SSTI, and it shows promise in the management of nocardiosis and ventilator-associated bacterial pneumonia.^4^ Notably, tedizolid exhibits a lower incidence of myelotoxicity and neurotoxicity compared with linezolid, a feature that may be particularly advantageous in prolonged treatment courses.^5–7^

Linezolid exhibits extensive tissue diffusion, including a high penetration coefficient in the cerebrospinal fluid (CSF), defined as the ratio of the AUC in the CSF to that in serum.^8^ This favourable pharmacokinetic profile enables linezolid to achieve CSF concentrations that ideally exceed the MICs of pathogens involved in neuromeningeal infections, rendering it a promising therapeutic agent.^9^

Tedizolid exhibits excellent oral bioavailability. In healthy subjects, oral administration of 200 mg resulted in a mean C_max_ of 2.0 mg/L and an AUC of 23.4 µg × h/mL, with systemic exposure increasing linearly across doses ranging from 200 to 600 mg.^10^ In a murine model of *Staphylococcus aureus* infection, the AUC/MIC ratio was identified as the pharmacokinetic/pharmacodynamic parameter most closely associated with therapeutic efficacy.^11^ Following intravenous infusion of 200 mg in healthy individuals, C_max_ values ranged from 1.86 to 3.89 mg/L, demonstrating systemic exposures comparable to those observed with oral dosing.^12^

However, *in vitro* data on tedizolid penetration into neuromeningeal tissues remain limited. Several studies have indicated poor CSF penetration, with reported penetration ratios ranging from 1.45% to 3.84%, indicating suboptimal entry of tedizolid into the CSF.^8,13^ These values appear insufficient to achieve concentrations exceeding the MICs of target pathogens. Moreover, few clinical studies have evaluated the limited neuro-meningeal diffusion of tedizolid in CSF.^14^

We present the case of a patient treated for skull base osteomyelitis complicated by acute aseptic meningitis, accompanied by a brief review of the relevant literature.

## Case presentation

A female in her sixties was admitted to the Emergency Department of the University Hospital of Réunion Island on December 2022, with fever, headache, and diplopia.

Her medical history was notable for a chronic external osteitis of the left ear. In 2014, she was diagnosed with external otitis complicated by osteitis of the left ear. Bone biopsies revealed methicillin-sensitive *Staphylococcus aureus*, but no antibiotic treatment was administered. In 2015, she underwent surgical removal of a left-sided cholesteatoma with external auditory canalplasty. Although chronic osteitis was suspected, it remained untreated. In December 2022, a cone beam CT scan of the left petrous bone showed multiple osseous defects in the left mastoid, consistent with chronic osteitis.

Upon admission, the patient reported having experienced vertigo for the past several months. Clinical examination revealed neck stiffness, without altered consciousness or focal neurological deficits. Although she complained of diplopia, no objective signs of oculomotor paralysis were observed. A CT scan of the brain was performed prior to lumbar puncture and revealed no evidence of intracranial hypertension. The LP demonstrated a normal opening pressure and marked hypercellularity, with 860 leukocytes/mm³ comprising 42% neutrophils and 47% lymphocytes. CSF analysis revealed elevated protein levels at 1.05 g/L (expected range: 0.15–0.4 g/L) and an increased lactate concentration of 3.9 mmol/L (expected range: 1.1–2.4 mmol/L). Glucose levels were within normal limits at 0.45 g/L, with a corresponding blood glucose level of 0.92 g/L. Direct Gram staining of the CSF was unremarkable. Empirical treatment for suspected bacterial meningitis was initiated, consisting of intravenous cefotaxime (300 mg/kg/day), intravenous amoxicillin (200 mg/kg/day), and dexamethasone (10 mg four times daily). The patient’s body mass index was 20.4. Renal function was preserved, with an estimated glomerular filtration rate of 97 mL/min/1.73 m^2^ (MDRD formula).

Subsequently, the patient was admitted to the infectious diseases ward (day 0). Emergency MRI of the brain and petrous bone revealed osteitis with a mesotympanic osteodural collection, suggestive of a residual cholesteatoma, accompanied by perilymphatic and subarachnoid fistulisations posterior to the acoustic-facial bundle, along with a 5 × 4 × 4 mm empyema and radiological features suspicious for leptomeningitis. Amoxicillin was promptly discontinued due to the absence of clinical or microbiological evidence supporting neurolisteriosis. CSF culture and all pathogen-specific PCR assays returned negative results, necessitating further investigation with broad-range 16S rRNA gene PCR, which was also negative.

Our final diagnosis was chronic otogenic skull base osteomyelitis on the left side, complicated by aseptic meningitis. In light of prior microbiological documentation of MSSA and the high prevalence of *Pseudomonas aeruginosa* in such infections, the antibiotic regimen was modified on day 2 to include intravenous ceftazidime (3 g three times daily) and oral tedizolid (200 mg once daily). Tedizolid was selected over linezolid due to its lower risk of myelosuppression, particularly in a planned 6-week treatment course. Uncertainty regarding CSF penetration was considered acceptable given the predominant involvement of bone. Emergency surgical intervention was not feasible at that time.

To assess CNS penetration of tedizolid, a repeat LP was performed on day 6 to evaluate cellular response and measure tedizolid levels in the CSF, 4 days after treatment initiation, corresponding to the steady state. CSF analysis demonstrated a leukocyte count decrease to 730 cells/mm^3^, with persistent elevation in protein concentration at 0.55 g/L and normalisation of lactate levels to 1.8 mmol/L. Simultaneous plasma and CSF samples were collected 24 h after the last tedizolid dose to determine trough concentrations in both compartments. These samples were analysed 3 days later at Bichat Hospital in Paris using liquid chromatography coupled with tandem mass spectrometry (LC-MS/MS; Xevo TQD; Waters, Milford, MA, USA), with a limit of quantification (LoQ) for tedizolid of 0.1 mg/L. The measured trough plasma concentration of tedizolid was 0.3 mg/L (expected range: 0.1–0.8 mg/L), whereas the CSF concentration was below the LoQ (Table 1).^15^ Despite these findings, tedizolid therapy was continued based on the patient’s clinical improvement, normalisation of inflammatory markers, and favourable CSF evolution. Following an antibiotic washout period, the patient underwent otolaryngologic surgery, including petrous bone milling and closure of the labyrinthine fistula. Intraoperative bone cultures were sterile. At follow-up 3 months after the completion of medical and surgical treatment, the patient remained clinically cured.

**Table 1.** Summary of studies that investigated the CSF penetration of tedizolid

Study	Micro-organisms	Other treatments	Tedizolid dose	CSF concentration (hours since last dose)	Plasma concentration (hours since last dose)	Mean penetration ratio	Outcome
Bouet et al.^14^ (patient 1)	*Nocardia farcinica* (MIC TZD 0.75 mg/L)	SXT/IMI/AMK	200 b.i.d. (oral)	0.11 mg/L (5)	1.494 mg/L (5)	20%	Death at 12 months
Bouet et al.^14^ (patient 2)	*Nocardia farcinica* (MIC TZD 0.75 mg/L)	SXT	200 b.i.d. (oral)	0.21 mg/L (10)	1 mg/L (10)	16%–27%	Living at 12 months
Wenzler et al.^16^	*Enterococcus faecium* (VRE)	Oritavancin	200 mg b.i.d. (IV)	0.204 mg/L (8.5)	0.30–0.49 mg/L (estimated)	54.8%	Death at 3 months
Matin et al.^17^	*Nocardia farcinica*	SXT	200 mg q.d. (oral)	Not done	Not done	Not done	Living at 12 months
Our study	Not documented	Ceftazidime	200 mg q.d. (oral)	< q.d. 0.1 mg/L (24)	0.3 mg/L (24)	<33%	Living at 6 months

AMK, amikacin; b.i.d., twice per day; CSF, cerebrospinal fluid; IMI, imipenem; IV, intravenous; MIC, minimum inhibitory concentration; q.d., once per day; SXT, sulfamethoxazole/trimethoprim; TZD, tedizolid; VRE, vancomycin-resistant enterococcus.

## Discussion

Linezolid has emerged as a first-line antibiotic for neuromeningeal infections caused by Gram-positive cocci, due to its excellent oral bioavailability and effective CNS penetration.^9,12^ This development has naturally raised interest in tedizolid, a newer oxazolidinone, as a potential alternative.

However, data on the CNS penetration of tedizolid remain limited. Moreover, clinical studies are scarce and primarily limited to a few case reports (Table 1). One such case reported a CSF concentration of 0.204 mg/L, corresponding to an estimated penetration ratio of approximately 50% relative to the plasma concentration.^17^ Notably, the CSF sample was obtained 8.5 h after the last intravenous administration of 200 mg of tedizolid (not a residual concentration), and the plasma concentration was not directly measured but extrapolated from estimated pharmacokinetic parameters. In particular, a C_max_ of 0.6 mg/L was used instead of the more accurate 2.0 mg/L, leading to an overestimation of the penetration ratio. In contrast, we report assays performed at the recommended sampling time, under real-life clinical conditions, using a widely validated method for therapeutic drug monitoring.

In another study, the tedizolid dose was increased to 200 mg twice daily in an attempt to overcome limited CNS penetration.^14^ Despite the penetration ratio remaining low (16%–27%), clinical outcomes were favourable.

Several case reports have demonstrated that CSF concentrations of tedizolid measured between 5 and 10 h after administration were < 0.22 mg/L. According to the EUCAST, most Gram-positive cocci are susceptible to tedizolid at an MIC of < 1 mg/L. Consequently, there is a substantial risk that therapeutic CSF concentrations may fall below the MIC for a significant portion of the dosing interval. Therefore, tedizolid appears to be an unsuitable candidate for the treatment of CNS infections.^18^

The pathophysiological rationale underlying the significantly lower CSF penetration of tedizolid compared with linezolid may be attributed to its higher protein binding, approximately 80% versus 30% for linezolid, which likely restricts its diffusion across the blood–brain barrier.^1^ Moreover, tedizolid is a substrate for efflux transporters such as P-glycoprotein and breast cancer resistance protein. *In vitro* studies have demonstrated that inhibition of these transporters results in a modest increase in tedizolid concentrations within the CSF.^14^ Variability in the affinity of tedizolid and linezolid for these efflux pumps and interindividual differences in transporter expression or activity may contribute to the observed differences in drug levels during sampling. In addition, the impact of meningeal inflammation on tedizolid penetration has not been systematically investigated.

A limitation of our case report is the LoQ, which may have hindered the detection of trough tedizolid concentrations due to insufficient assay sensitivity. However, even under the most optimistic assumptions (CSF concentration of approximately 0.09 mg/L), the CSF-to-serum concentration ratio would remain around 30%. In addition, only residual tedizolid concentrations were available for analysis, whereas the AUC_CSF_/AUC_plasma_ is the most reliable parameter for evaluating CSF penetration. However, determining this ratio necessitates repeated sampling, which is impractical in most clinical contexts, except in patients with a ventriculostomy.

Data concerning tedizolid pharmacokinetics, particularly its CNS penetration, are limited and heterogeneous. Consequently, further studies are needed to determine whether tedizolid is appropriate for the treatment of CNS infections. In addition, the need for dose-escalation strategies should be assessed in future studies.

### Conclusion

Tedizolid may be suboptimal for the treatment of CNS infections due to its limited CSF penetration at standard dosing. Compared with tedizolid, linezolid demonstrates more favourable pharmacokinetics for CNS infection management and should be preferred.

## Data Availability

De-identified data will be shared with other researchers upon reasonable request to the corresponding author (xavier.brousse@chu-bordeaux.fr). The sharing of data will require a detailed proposal submitted to the study investigators, and a data transfer agreement must be signed.
